# The Physician’s Hand Book for 1882

**Published:** 1881-11-20

**Authors:** 


THE Physician's Hand Book for 1882, by Wm. Elmer, M. D., and Albert D. Elmer, M. D., New York, W. A. Townsend, Publisher, contains special classifications, poisons, tests for urine, incompatibles, weights and measures, abbreviations, doses, materia medica, extemporaneous prescriptions, ample space for record of visits, memoranda, etc., etc.

